# Bridging Lifestyle and Screening for Cancer Prevention: A Comprehensive Analysis of Cancer-Related Lifestyle and Screening Attitudes in Adults

**DOI:** 10.3390/medicina61030510

**Published:** 2025-03-16

**Authors:** Arda Borlu, Halime Şentürk, Hasan Durmuş, Neslihan Öner, Ebru Tan, Umut Köleniş, Müncübe Duman Erbakırcı, Fevziye Çetinkaya

**Affiliations:** 1 Department of Public Health, Faculty of Medicine, Erciyes University, 38280 Kayseri, Türkiye; ardab@erciyes.edu.tr (A.B.); halimesenturk@erciyes.edu.tr (H.Ş.); hasandurmus@erciyes.edu.tr (H.D.); ebrutan@erciyes.edu.tr (E.T.); fevcetin@erciyes.edu.tr (F.Ç.); 2 Department of Nutrition and Dietetics, Faculty of Health Sciences, Erciyes University, 38280 Kayseri, Türkiye; 3 Niğde Ömer Halisdemir Training and Research Hospital, 51100 Niğde, Türkiye; umut.kolenis@saglik.gov.tr; 4 İncesu Ayşe ve Saffet Arslan School of Health Services Vocational Studies, 38280 Kayseri, Türkiye; muncubeerbakirci@kayseri.edu.tr

**Keywords:** cancer prevention, lifestyle behaviors, cancer screening, public health, Türkiye

## Abstract

*Background and Objectives:* Healthy lifestyle behaviors and cancer screening are crucial for cancer prevention; however, their relationship remains inadequately explored. This study examines cancer-related lifestyle behaviors, attitudes toward cancer screening, and their interrelationship in adults. *Materials and Methods:* A descriptive cross-sectional study was conducted among 1129 adults (aged 18–70) visiting family health centers in Kayseri, Türkiye. Data was collected via face-to-face surveys assessing sociodemographic characteristics, lifestyle behaviors (Lifestyle Questionnaire Related to Cancer), and screening attitudes (Attitude Scale Toward Cancer Screenings). Statistical analyses included Mann–Whitney U, Kruskal–Wallis, and Spearman correlation tests. *Results:* Women, married participants, urban residents, and those with higher education and income exhibited healthier lifestyle behaviors and more positive attitudes toward cancer screening (*p* < 0.05). A weak but significant correlation (r = 0.247, *p* < 0.05) was found between healthy lifestyle behaviors and positive screening attitudes. Despite national screening programs, adherence to breast and cervical cancer screenings remained low (51.5% and 44.6%, respectively). Having a first-degree relative with cancer did not significantly influence screening behaviors. *Conclusions:* Gender, education, income, and marital status significantly influence cancer prevention behaviors. However, screening participation remains suboptimal, highlighting the need for targeted public health strategies. Improving health literacy and accessibility to screening programs could enhance cancer prevention efforts.

## 1. Introduction

Cancer is one of the most significant global public health challenges, responsible for approximately 10 million deaths in 2020 (WHO). Nearly one in every six deaths is directly attributable to cancer or its related complications. In terms of the overall health burden—measured by disability-adjusted life years (DALYs) and years of life lost (YLL)—cancer ranks second only to cardiovascular diseases [1]. The World Health Organization (WHO) projects that the number of cancer cases will double by 2030, positioning cancer as the leading cause of death worldwide [2]. Beyond its mortality toll, the disease imposes profound emotional, physical, and economic burdens on patients, their families, and healthcare systems.

Among the most prevalent cancer types globally are prostate cancer in men and breast and cervical cancers in women, as well as colorectal, lung, stomach, and skin cancers in both sexes. Collectively, these malignancies account for nearly half of all new cancer cases [3]. Many of these cancers are strongly associated with modifiable lifestyle factors [4,5]. Although the etiologies of cancer are multifactorial and complex, prevention through lifestyle modification remains one of the most cost-effective strategies to combat disease [2].

A variety of behavioral risk factors have been implicated in cancer etiology, including tobacco exposure, alcohol consumption, physical inactivity, unhealthy dietary habits, obesity, and certain infections [6]. The WHO emphasizes that approximately 30–50% of cancers could be prevented by avoiding these risk factors and implementing primary prevention strategies [2]. At both the individual and community levels, promoting a healthy lifestyle is crucial for cancer prevention [7]. In support of this approach, several studies have demonstrated that regular physical activity and exercise can lower cancer-related morbidity and mortality. At the same time, a diet characterized by high nutritional quality is associated with a reduced risk of developing cancer [8].

Early detection through cancer screening is another key to cancer prevention [9]. Screening tests enable the detection of malignancies before the onset of symptoms, thereby facilitating early diagnosis and intervention, which in turn can significantly reduce mortality [10,11,12,13]. For example, modern mammography programs have been shown to reduce breast cancer mortality by more than 40% [14,15]. A model-based cohort study by Sharma et al. estimated that—if current screening levels were maintained—approximately 10,179 breast cancer deaths, 27,166 cervical cancer deaths, and 74,740 colorectal cancer deaths could be prevented [16].

In Türkiye, national screening guidelines recommend the following: for colorectal cancer, biennial fecal occult blood testing (FOBT) in individuals aged 50–70 years with a colonoscopy every 10 years; for breast cancer, self-breast examination, an annual clinical breast examination, and biennial mammography for women aged 40–69 years; and for cervical cancer, a Pap smear together with HPV DNA testing every 5 years for women aged 30–65 years [12]. There is an ongoing debate about the effectiveness of breast self-examination and clinical breast examination in reducing mortality, and many international organizations no longer recommend them as screening methods [17]. Participation rates remain low despite these recommendations and the availability of free screening services in public healthcare facilities. For instance, less than 25% of Turkish women aged 50–69 have undergone mammography within two years, a rate that is markedly lower compared to many OECD countries. In 2021, cervical cancer screening rates were around 33%, and colorectal cancer screening rates were approximately 9% in Türkiye [11]. Evidence-based, multifaceted interventions have improved screening uptake, especially among populations with historically low participation [13]. It is also well-documented that individuals with low income or without health insurance tend to have lower screening rates [18,19]. However, merely providing insurance may not be sufficient; understanding cultural and other sociodemographic factors is critical to addressing these disparities.

Moreover, studies investigating attitudes toward cancer screenings have found that greater knowledge about screening methods is associated with more positive attitudes toward participation [20]. In addition, research among healthcare workers indicates that having a personal or family history of cancer increases the likelihood of undergoing regular screening tests [21].

Therefore, healthy lifestyle behaviors and cancer screening are two key cancer prevention approaches. This study evaluates cancer-related lifestyle behaviors and attitudes toward cancer screenings, examining their interrelationship in adults as key components of cancer prevention. Unlike many studies in the literature that have examined cancer prevention approaches concerning a specific cancer type or a particular sociodemographic characteristic, this study provides a comprehensive overview of the cancer-related lifestyles and general attitudes toward cancer screenings across the entire adult population. The insights gained are expected to contribute to the development of more effective public health strategies for cancer prevention.

## 2. Materials and Methods

### 2.1. Study Population and Sampling Method

This descriptive cross-sectional study targeted literate adults aged 18–70 who visited family health centers in central Kayseri for any reason. The sample consisted of participants who visited these centers between May and June 2024, volunteered to participate, and had no conditions preventing them from answering the survey questions. The minimum sample size was determined using G*Power 3.1.9.7 for a bivariate normal model, assuming a small effect size with a two-tailed α = 0.05 and β = 0.90; the calculation yielded a minimum requirement of 1046 participants. Ultimately, the study was completed with a total of 1129 participants.

### 2.2. Data Collection Instruments

The survey was conducted through face-to-face interviews conducted by the researchers after providing detailed information about the study and obtaining verbal consent from each participant. Data was collected using a survey form consisting of three sections:

- Section one: This section of the survey, prepared by the researchers, included questions designed to capture participants’ sociodemographic characteristics, their perceived general health status, and their attitudes and behaviors related to cancer.
- Section two: This section utilized the “Cancer-Related Lifestyle Scale” (LQ-RC) developed by Momayyezi et al. [22] and validated in Turkish by Öner et al. [23]. The scale consists of 37 items grouped into eight sub-dimensions and employs a 4-point Likert-type response format. All items are scored as follows: 0 = never, 1 = sometimes, 2 = usually, and 3 = always, with no items requiring reverse scoring. The total score ranges from 0 to 111, with a Cronbach’s alpha of 0.881. The eight sub-dimensions are: “Stress Management”, “Avoidance of Risky Eating Behaviors”, “Utilization of Preventive Health Services”, “Physical Health Status”, “Physical Activity and Exercise”, “Sufficient and Balanced Nutrition”, “Avoidance of Substance Use”, and “Risk-Reducing Practices”.
- Section three: This section featured the “Attitude Scale Toward Cancer Screenings (ASTCS)”, which has undergone validity and reliability testing in Turkish [24]. The scale comprises 24 items forming a single-dimensional structure and uses a 5-point Likert-type scale with response options defined as follows: 5 = completely agree, 4 = partly agree, 3 = neither agree nor disagree, 2 = partly disagree, and 1 = completely disagree. Total scores range from 24 to 120; scores near 24 indicate a negative attitude toward cancer screening, while scores near 120 indicate a positive attitude. Thirteen items are reverse-scored, and the scale’s Cronbach’s alpha is 0.950.

### 2.3. Statistical Analysis

The normality of the data was assessed using the Kolmogorov–Smirnov test, coefficient of variation (<0.20), Normal Q–Q Plot, detrended Q–Q Plot, and histogram analyses. Data analyses were conducted using IBM SPSS Statistics 28. Descriptive statistics were calculated for all variables. The Mann–Whitney U test was applied to compare two groups, while the Kruskal–Wallis test was used for comparisons among three or more groups. A *p*-value of <0.05 was considered statistically significant. Additionally, Spearman correlation analysis was performed to examine relationships between the scales.

### 2.4. Ethical Procedure

The study procedure was performed according to Declaration of Helsinki and STROBE checklist. The study was approved by the Social and Human Sciences Ethics Committee of Erciyes University (Date: 26 March 2024 and Approval No: 2024/159). Participants were informed about the study, and their verbal consent was obtained.

## 3. Results

The mean scores obtained by the participants on the scales are presented in Table 1. The mean ASTCS score was 88.93 ± 22.86. The total LQ-RC score was 60.59 ± 18.07 (range: 0–111).

The mean age of the participants was 37.15 ± 12.94 years. Of the participants, 57.9% were female, 59.2% were married, and 27% and 44.7% had at least a college/university degree. Additionally, 49.0% reported that their income and expenses were balanced, and 55.8% had children. The sociodemographic characteristics of the participants are presented in Table 2.

Comparison of participants’ characteristics based on the median score of the LQ-RC is presented in Table 2. When we evaluate the adherence of female participants to the recommended cancer screenings according to their age groups, among women in the 40–69 age group (n = 260), 51.5% had undergone at least one mammography and/or breast ultrasound. Additionally, among women in the 30–65 age group (n = 404), 44.6% had undergone at least one cervical smear test (Table 2). The median scores of the LQ-RC were found to be significantly higher among women compared to men, university graduates compared to those with a high school education or lower, participants living in urban areas compared to those in rural areas, married participants compared to single or divorced/widowed participants, those with children compared to those without, non-smokers compared to smokers, and those using dietary supplements compared to those who do not. No significant difference was found between age groups, having a first-degree relative with cancer, or BMI (Body Mass Index) and the median score of the LQ-RC.

Table 3 presents the comparison of participants’ characteristics based on the median ASTCS score. Women, university graduates (compared to those with a high school education or lower), married participants (compared to singles), and dietary supplement users (compared to non-users) had significantly higher scores.

**Figure 1:**
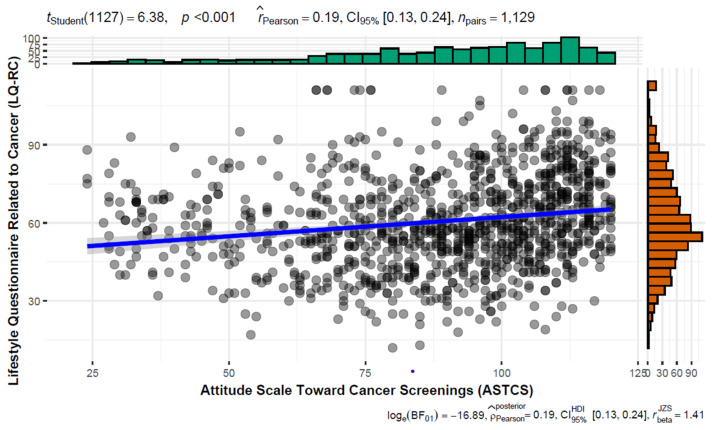
The median score of the LQ-RC and ASTCS. Note: The colors represent the following meaning. Green: ASTCS score, Orange: LQ-RC score, Blue: regression line.

Figure 1 presents the median score of the LQ-RC and ASTCS. The median score of the LQ-RC was 59.00 (Q1-Q3; 49.00–72.00), while the median score of the ASTCS was 94.00 (Q1-Q3; 75.00–108.00). A statistically significant positive, very weak correlation (r = 0.247) was found between the LQ-RC and ASTCS median scores (Figure 1).

## 4. Discussion

In this study, aimed at examining the sociodemographic determinants and relationships of two key cancer prevention approaches, lifestyle and screening, 1129 adults were interviewed. The mean ASTCS score for the group was 88.93 ± 22.86, while the median score for women was 96.00 (80.00–109.00). A comparison with other studies conducted in Türkiye using the same scale reveals variations in median scores across different study populations. In a study where the scale was developed and applied to an equal number of male and female participants, the total ASTCS score was reported as 60.51 ± 27.80 [24]. Another study among participants aged 30–70 in Türkiye found an average score of 101.6 ± 12.85% [25]. A study conducted exclusively among women in Türkiye, which used a short form of the ASTCS, reported a mean score of 65.19 ± 8.45 [20]. Similarly, a study assessing knowledge, attitudes, and behaviors regarding cancer screenings among municipal employees in a central district of Ankara found a median ASTCS score of 92.0 (52.0–120.0) [26]. Another study investigating women’s attitudes toward cancer screenings and related factors in Turkey reported an average ASTCS total score of 84.36 ± 14.55 [21]. In a study conducted in Türkiye, where data were collected online, the mean score of the participant’s attitude toward cancer screening was 94.57 ± 18.39 [27]. These findings underscore the variability in cancer screening attitudes across different populations, even in the same country. Although the study’s ASTCS scores are higher than those reported in some previous studies, they still fall short of the optimal levels required for widespread participation in cancer screenings. The observed differences between studies may be influenced by factors such as sample characteristics and accessibility of healthcare services.

Women had significantly higher total scores on both the ASTCS and LQ-RC, as well as in the subdimensions of LQ-RC; Avoidance of Risky Eating Behaviors, Utilization of Preventive Health Services, Avoidance of Harmful Substance Use, and Risk-Reducing Practices, compared to men. In Erkal’s study [27], like this study, women’s attitude scores toward cancer screening were higher. This suggests women exhibit more positive attitudes toward health screenings and a healthy lifestyle. The literature presents varying findings regarding gender differences in ASTCS scores. In the primary study where Öztürk et al. [24] ASTCS was developed, men demonstrated more positive attitudes toward cancer screening than women. However, studies by Yegenler et al. [25] and Bağcı et al. [26] found no statistically significant differences in ASTCS scores between genders. These inconsistencies suggest that gender-related variations in attitudes toward cancer screening may be influenced by demographic, cultural, or contextual factors, warranting further investigation. Consistent with our study, a systematic review investigating gender-specific differences in primary prevention behaviors concluded that women generally tend to engage in more and better preventive behaviors, except for physical activity [28]. This finding reinforces the idea that women are more proactive in adopting health-promoting behaviors, which may contribute to their lower cancer incidence rates than men. Additionally, a review emphasizing the role of gender in cancer development and treatment highlighted that, while not the sole determinant, the higher prevalence of risky lifestyle choices among men—such as tobacco and alcohol use, poor dietary habits, and lower engagement in preventive healthcare—is linked to the increased cancer incidence in males [29]. These gender differences underscore the need for public health interventions that address the specific barriers men face in adopting preventive behaviors. Efforts to increase male participation in cancer screenings and other preventive measures should incorporate targeted education, behavioral incentives, and community-based initiatives. Developing gender-sensitive health promotion strategies may help bridge the gap in preventive behaviors and contribute to reducing cancer incidence among men.

No significant relationship was found between having a first-degree relative diagnosed with cancer and ASTCS or LQ-CR scores. Supporting this finding, a study examining the impact of a first-degree relative’s cancer diagnosis on participants’ participation in cancer screening programs and attitudes toward healthy lifestyle changes similarly concluded that having a close relative with cancer did not serve as a sufficient motivator for participants to undergo cancer screening [30]. This suggests that personal experience with cancer in the family may not always translate into proactive health behaviors, highlighting the need for targeted awareness and education strategies. However, there are studies in the literature with differing results. For example, a study conducted in Saudi Arabia identified a positive family history of breast cancer as the only significant factor associated with a positive attitude toward mammography [31]. These discrepancies suggest that cultural, social, and healthcare system differences may play a role in shaping participants’ responses to cancer risk within their families.

The study revealed that married participants, those with education above high school level, those who use dietary supplements, and those with good economic status had higher LQ-RC and ASTCS scores. Although Erkal’s study [27] was consistent with this study in terms of lower ASTCS scores among those with low income and higher scores among university graduates, the higher ASTCS scores among single participants differed from this study. In line with the findings of this study, a study conducted on 28,047 adults in Norway revealed that men and participants with lower education levels are more likely to have health-related risks [32]. In the literature, numerous studies have identified low socioeconomic status as a barrier to participation in cancer screenings [33,34]. A knowledge, attitude, and practice (KAP) study on colorectal cancer screenings conducted in China found no association between attitudes toward cancer screenings and educational level or economic status [35]. This finding suggests that the impact of socioeconomic factors on screening attitudes may vary depending on cultural and structural dynamics.

In a study conducted in Saudi Arabia, it was highlighted that dietary supplement use is often associated with the adoption of other healthy habits, such as consuming a balanced diet, engaging in physical exercise, weight control, and non-smoking, as part of a healthier lifestyle [36]. This study found that participants residing in urban areas, those with children, non-smokers, and students had higher LQ-RC scores. In a study aiming to assess rural-urban differences in health-related quality of life for elderly cancer survivors and controls, similar to this study, health-related quality of life scores were higher in urban areas compared to rural areas [37]. In a study aiming to identify the distribution and associated factors of four healthy lifestyle behaviors in China (non-smoking, limited alcohol consumption, leisure-time physical activity, and a healthy diet) and their potential health benefits on mortality, it was generally found that participants who were female, lived in urban areas, had higher education, or had higher household income were more likely to adhere to all healthy lifestyle behaviors [38].

In this study, although ASTCS scores were higher among non-smokers, no significant association was found between smoking and adherence to cancer screenings. Unlike our study, a cohort study conducted exclusively among women found that active smoking was strongly associated with a decrease in the utilization of cancer screening services [39]. In the literature, some other studies have also shown that smokers are more likely to exhibit negative health behaviors related to cancer screening [40,41].

In the study, a positive but weak correlation was observed between ASTCS and LQ-RC scores, indicating that while healthy lifestyle behaviors are associated with positive attitudes toward cancer screenings, they are influenced by different underlying factors. This suggests that although adopting a healthy lifestyle may support more significant participation in cancer screenings, both aspects should be addressed through distinct yet complementary strategies.

Consistent with our findings, a study conducted in Türkiye among participants aged 50 and above who visited a family health center found no significant association between lifestyle behaviors and colorectal cancer screening participation [42]. However, in contrast, research conducted in Australia demonstrated that participants who adhered to a healthy lifestyle were more likely to have undergone colorectal cancer screening within the past two years. Furthermore, the existing literature indicates that participants with unhealthy lifestyles exhibit lower participation rates in screening programs and other preventive measures [43,44]. These findings highlight the potential influence of cultural and behavioral factors on screening adherence, underscoring the importance of tailored public health interventions to promote both healthy lifestyle behaviors and cancer screening participation effectively.

In the study, women’s adherence to the recommended cancer screenings according to their age groups was also evaluated; only 44.6% of them had undergone a cervical smear test. Eliminating cervical cancer, a major public health concern, requires reducing its incidence to 4 per 100,000 women or lower. To achieve this, 70% of women aged 35–45 should undergo screening with a high-quality diagnostic test [2]. However, a study conducted among participants visiting family medicine clinics found that the cervical smear test rate was only 31.8% [45]. Globally, it is estimated that only 36% of women aged 30–49 have undergone cervical cancer screening at least once in their lifetime [46]. These findings, including those from the study, indicate that the targeted screening levels for cervical cancer elimination have yet to be met. In the study, women who underwent cervical cancer screening had more positive attitudes toward cancer screenings. A study assessing the knowledge, attitudes, and practices regarding cervical cancer screening among female university students in Western Uganda found a weak correlation between attitudes toward cervical cancer screening and knowledge about participation in screening programs [47]. A systematic review and meta-analysis in Ethiopia found that women’s attitudes significantly influenced cervical cancer screening uptake [48]. These findings suggest that positive attitudes toward cervical cancer screening can encourage participation in cancer screening programs.

The rate of women undergoing mammography/breast ultrasound for breast cancer screening was 51.5% at the time of the study. When comparing mammography rates worldwide and in Türkiye, Denmark has achieved a high screening rate of 83% among the target population, whereas in Türkiye, fewer than 25% of women in the target age group have had a mammogram in the past two years [11]. Although the study results exceed the national average, it is evident that screening rates remain insufficient. A study conducted in Iran found a positive correlation between women’s breast cancer screening behaviors and their attitudes [49]. Similarly, although not statistically significant, the study also found that women who underwent breast cancer screening had higher scores on the attitude scale toward cancer screenings.

## 5. Limitations

The data used in this study are based on participants’ self-reports, which may have introduced social bias. Additionally, due to the cross-sectional study design, establishing causal relationships was not possible. Furthermore, the regional characteristics of the study population and variations in access to healthcare services may limit the generalizability of the findings.

## 6. Conclusions

This study provides insights into the sociodemographic determinants influencing attitudes and behaviors toward two key cancer prevention approaches, including lifestyle choices and screening participation. The findings highlight significant gender differences, with women exhibiting more positive attitudes toward cancer screenings and healthier lifestyle behaviors compared to men. While consistent with some studies, these differences are not universally observed, suggesting the potential influence of cultural and demographic factors. The study also underscores the role of socioeconomic status, education level, marital status, and urban residency in shaping attitudes toward cancer screening and preventive health behaviors.

The lack of a significant relationship between having a first-degree relative with cancer and screening attitudes challenges the assumption that personal exposure to cancer risk automatically translates into proactive health behaviors. This finding, alongside inconsistencies in the literature, underscores the necessity for targeted awareness campaigns to address potential psychological and social barriers to screening participation.

Although the study identified a positive but weak correlation between attitudes toward cancer screening and healthy lifestyle behaviors, the findings suggest that these two dimensions are influenced by different underlying factors. Therefore, public health strategies should address them through distinct yet complementary interventions. One of the most concerning findings of the study is the suboptimal adherence to cervical and breast cancer screening recommendations among women. In Türkiye, these screenings are provided free of charge by the government, yet participation rates remain low. These findings underscore the necessity for improved public health strategies, education campaigns, and policy measures to enhance participation in both cervical and breast cancer screening programs.

Overall, this study emphasizes the importance of sociodemographic factors in shaping cancer prevention behaviors and highlights the need for gender-sensitive, socioeconomic status-informed, and culturally tailored interventions. Future research should further explore the underlying factors influencing disparities in cancer screening participation and lifestyle behaviors to develop more effective public health strategies.

## Figures and Tables

**Table 1:** Mean scores of participants on the Attitude Scale Toward Cancer Screenings (ASTCS) and the Cancer-Related Lifestyle Scale (LQ-RC) and its sub-dimensions.

Means	X ± SD	Min–Max
Attitude Scale Toward Cancer Screenings (ASTCS)	88.93 ± 22.86	24–120
Cancer-Related Lifestyle Scale Total (LQ-RC)	60.59 ± 18.07	0–111
LQ-RC—Stress Management	15.79 ± 4.90	0–24
LQ-RC—Avoidance of Risky Eating Behaviors	7.92 ± 3.67	0–15
LQ-RC—Utilization of Preventive Health Services	6.33 ± 3.80	0–15
LQ-RC—Physical Health Status	9.52 ± 3.46	0–15
LQ-RC—Physical Activity and Exercise	5.29 ± 3.24	0–12
LQ-RC—Sufficient and Balanced Nutrition	5.41 ± 2.88	0–12
LQ-RC—Avoidance of Substance Use	5.78 ± 2.42	0–9
LQ-RC—Risk-Reducing Practices	4.56 ± 2.62	0–9

**Table 2:** Comparison of participants’ characteristics based on the median score of the LQ-RC.

Characteristics	n (%)	Median (Q1–Q3)	z	*p*
Gender				
Male	475 (42.1%)	57.00 (46.00–71.00)	2.377	0.017
Female	654 (57.9%)	60.00 (50.00–73.00)		
Age				
18–29 years	419 (37.1%)	57.00 (48.00–71.00)	3.780	0.286
30–39 years	257 (22.8%)	61.00 (47.50–71.50)		
40–64 years	423 (37.5%)	60.00 (50.00–74.00)		
≥65 years	30 (2.6%)	59.00 (45.25–77.75)		
Education level				
Primary school or below (a)	189 (16.8%)	55.00 (46.50–68.50)	13.114	0.001
Secondary School–High School (a)	435 (38.5%)	58.00 (48.00–71.00)		
College–University (b)	505 (44.7%)	61.00 (51.00–74.00)		
Longest-term residence				
Urban	1041 (92.2%)	60.00 (49.00–73.00)	4.174	≤0.001
Rural	88 (7.8%)	52.00 (41.00–62.75)		
Marital status				
Married	668 (59.2%)	60.00 (50.00–74.00)	2.329	0.020
Single	461 (40.8%)	57.00 (46.50–70.00)		
Having children				
Yes	630 (55.8%)	60.50 (50.00–73.00)	2.254	0.024
No	499 (44.2%)	57.00 (47.00–71.00)		
Economic status				
Income < Expenses (a)	334 (29.6%)	56.00 (43.75–69.00)	19.180	≤0.001
Income = Expenses (b)	553 (49.0%)	59.00 (50.00–72.50)		
Income > Expenses (b)	242 (21.4%)	61.50 (52.00–77.00)		
Smoking status				
Smoker	333 (29.5%)	57.00 (47.00–69.00)	2.941	0.003
Non-smoker	796 (70.5%)	60.00 (50.00–74.00)		
First-degree family history of cancer				
Yes	244 (21.6%)	60.00 (50.25–71.00)	0.435	0.664
No	885 (78.4%)	59.00 (48.00–72.00)		
Use of dietary supplements				
Yes	129 (11.4%)	63.00 (53.00–77.00)	3.222	0.001
No	1000 (88.6%)	59.00 (48.00–71.00)		
BMI classification				
Underweight	40 (3.5%)	59.50 (48.50–68.00)	2.923	0.404
Normal	503 (44.6%)	59.00 (49.00–73.00)		
Overweight	408 (36.1%)	59.50 (50.00–72.75)		
Obese	178 (15.8%)	56.00 (47.00–71.00)		
Mammography/breast ultrasound (n = 260)				
Yes	134 (51.5%)	64.00 (54.75–77.00)	2.309	0.021
No	126 (48.5%)	58.00 (50.00–72.00)		
Cervical smear test (n = 404)				
Yes	180 (44.6%)	64.00 (55.25–72.25)	1.747	0.081
No	224 (55.4%)	60.00 (48.00–76.00)		

**Table 3:** Comparison of participants’ characteristics based on the median score of the ASTCS.

n = 1129	n (%)	Median (Q1–Q3)	z	*p*
Gender				
Male	475 (42.1)	89.00 (72.00–106.00)		
Female	654 (57.9)	96.00 (80.00–109.00)	3.375	≤0.001
Age				
18–29 years	419 (37.1)	95.00 (75.00–107.00)	4.785	0.118
30–39 years	257 (22.8)	91.00 (74.00–107.00)		
40–64 years	423 (37.5)	96.00 (76.00–109.00)		
≥65 years	30 (2.6)	90.00 (72.00–102.00)		
Education level				
Elementary and below	189 (16.8)	92.00 (75.50–103.50)	11.569	0.003
Middle school–High school	435 (38.5)	90.00 (72.00–106.00)		
College–University	505 (44.7)	97.00 (78.00–109.00)		
Longest-term residence				
Urban	1041 (92.2)	94.00 (75.00–108.00)	0.170	0.865
Rural	88 (7.8)	90.50 (75.25–109.00)		
Marital status				
Married	668 (59.2)	96.00 (76.00–109.00)	2.648	0.008
Single	461 (40.8)	92.00 (73.50–105.00)		
Having children				
Yes	630 (55.8)	95.00 (76.00–108.00)	1.940	0.052
No	499 (44.2)	93.00 (73.00–106.00)		
Economic status				
Income < Expenditure	334 (29.6)	92.00 (74.75–106.00)	7.325	0.026
Income = Expenditure	553 (49.0)	94.00 (75.00–107.00)		
Income > Expenditure	242 (21.4)	99.00 (77.00–110.00)		
Smoking status				
Smoker	333 (29.5)	92.00 (71.50–106.50)	1.551	0.121
Non-smoker	796 (70.5)	95.00 (76.00–108.00)		
First-Degree relatives with cancer				
Yes	244 (21.6)	94.50 (75.00–108.00)	0.353	0.724
No	885 (78.4)	94.00 (75.50–108.00)		
Use of dietary supplements				
Yes	129 (11.4)	98.00 (78.00–110.50)	2.077	0.038
No	1000 (88.6)	94.00 (75.00–107.00)		
BMI classification				
Underweight	40 (3.5)	95.50 (70.75–105.00)	2.865	0.413
Normal	503 (44.6)	93.00 (75.00–106.00)		
Overweight	408 (36.1)	96.00 (76.00–109.00)		
Obese	178 (15.8)	94.50 (74.75–107.25)		
Mammography/Breast ultrasound (n = 260)				
Yes	134 (51.5)	101.50 (77.00–111.25)	1.914	0.056
No	126 (48.5)	93.00 (78.00–105.25)		
Cervical smear (n = 404)				
Yes	180 (44.6)	102.00 (85.00–112.00)	4.010	≤0.001
No	224 (55.4)	92.00 (73.25–106.00)		

## Data Availability

All data generated or analyzed during this study are included in this article. Further enquiries can be directed to the corresponding author.
